# Oral Supplement Containing Hydroxytyrosol and Punicalagin Improves Dyslipidemia in an Adult Population without Co-Adjuvant Treatment: A Randomized, Double-Blind, Controlled and Crossover Trial

**DOI:** 10.3390/nu14091879

**Published:** 2022-04-29

**Authors:** Rebeca Quirós-Fernández, Bricia López-Plaza, Laura M. Bermejo, Samara Palma Milla, Andrea Zangara, Carmen Gómez Candela

**Affiliations:** 1 Nutrition Research Group, Hospital La Paz Institute for Health Research (IdiPAZ), 28046 Madrid, Spain; mlbermej@ucm.es; 2 Nutrition Department, Hospital University La Paz, 28046 Madrid, Spain; samara.palma@salud.madrid.org (S.P.M.); cgcandela@salud.madrid.org (C.G.C.); 3 Centre for Human Psychopharmacology, Swinburne University, Melbourne, VIC 3122, Australia; azangara@euromed.es; 4 Euromed S.A., C/Rec de Dalt, 21-23, Pol. Ind. Can Magarola, 08100 Mollet del Valles, Spain

**Keywords:** cardiovascular disease, atherosclerosis, hydroxytyrosol, punicalagin, dyslipidemia, total cholesterol, low-density lipoprotein cholesterol, high-density lipoprotein cholesterol, triglycerides

## Abstract

Hydroxytyrosol (HT) and punicalagin (PC) exert cardioprotective and antiatherosclerotic effects. This study evaluated the effect of an oral supplement containing HT and PC (SAx) on dyslipidemia in an adult population. A randomized, double-blind, controlled, crossover trial was conducted over a 20-week period. SAx significantly reduced the plasma levels of triglycerides (TG) in subjects with hypertriglyceridemia (≥150 mg/dL) (from 200.67 ± 51.38 to 155.33 ± 42.44 mg/dL; *p* < 0.05), while no such effects were observed in these subjects after the placebo. SAx also significantly decreased the plasma levels of low-density lipoprotein cholesterol (LDL-C) in subjects with high plasma levels of LDL-C (≥160 mg/dL) (from 179.13 ± 16.18 to 162.93 ± 27.05 mg/dL; *p* < 0.01), while no such positive effect was observed with the placebo. In addition, the placebo significantly reduced the plasma levels of high-density lipoprotein cholesterol (HDL-C) in the total population (from 64.49 ± 12.65 to 62.55 ± 11.57 mg/dL; *p* < 0.05), while SAx significantly increased the plasma levels of HDL-C in subjects with low plasma levels of HDL-C (<50 mg/dL) (from 44.25 ± 3.99 to 48.00 ± 7.27 mg/dL; *p* < 0.05). In conclusion, the supplement containing HT and PC exerted antiatherosclerotic and cardio-protective effects by considerably improving dyslipidemia in an adult population, without co-adjuvant treatment or adverse effects.

## 1. Introduction

Cardiovascular diseases (CVDs) remain the leading cause of disease burden in the world. An estimated 17.9 million people died from CVDs in 2019, representing 32% of all global deaths [1]. The recommendation by the World Health Organization (WHO) to help reduce the global burden of CVD is designed to provide counseling and adequate treatment for at least 50% of eligible people (defined) as aged 40 years or older and at high risk of CVD) by 2025 [2]. People considered to be at high risk for CVD are those with one or more risk factors, such as dyslipidemia, arterial hypertension, diabetes, or previously established disease [3]. To reduce the global burden of CVD, early detection and primary prevention are essential [3], and the WHO considers subjects aged 40–80 years, without a known baseline history of CVD, to be the target population in primary prevention efforts [2].

It has been widely recognized that most CVDs can be prevented by addressing behavioral risk factors, such as unhealthy diet, physical inactivity, and harmful tobacco and alcohol use, as well as appropriate control of risk conditions for CVDs, including dyslipidemia, arterial hypertension, diabetes and obesity [4]. Although the remarkable success of pharmacotherapy and preventive efforts have been introduced in the past decades, CVDS still constitutes a public health challenge as a top cause of morbidity, loss of useful life years, and mortality worldwide [1]. Therefore, any efforts for the prevention of CVD should be strongly encouraged. 

Atherosclerosis, otherwise known as an atherosclerotic vascular disease (ASVD), is the main cause of mortality in CVD [5]. In ASVD, the buildup of plaques within blood vessels, resulting in the restriction of blood flow, with a potential risk of rupture, contributes to the development of heart attacks (myocardial infarction) and strokes, which can be fatal [6].

Dyslipidemia is one of the major risk factors for the development and progression of ASVD and CVDs [7,8]. Dyslipidemia includes a wide range of lipid abnormalities and may involve a combination of increased plasma levels of total cholesterol (TC), low-density lipoprotein cholesterol (LDL-C), and triglycerides (TG), or decreased high-density lipoprotein cholesterol (HDL-C). The prevention and sensible management of dyslipidemia can positively modify CV morbimortality [8]. Therefore, an effective solution with few or no adverse effects and high adherence, could reduce the ASVD morbimortality and, CVDs.

Polyphenols are becoming increasingly accepted as therapeutic substances for addressing a wide range of diseases, such as ASVD and CVDs [9,10,11,12,13,14,15,16,17,18,19,20,21,22], and their risk factors [9,11,12,13,14,15,16,17,20,21,22,23,24,25,26]. Diverse studies have reported an inverse correlation between polyphenol consumption and the risk of CV events [10,18,23,27] and overall mortality [23,28,29,30]. Among these bioactive compounds, hydroxytyrosol (HT), from olives, and punicalagin (PC), from pomegranates, are noteworthy for their antioxidant, antiatherosclerotic, cardioprotective, neuroprotective, anticancer, and other effects [13,26,31,32,33,34,35,36]; in this article, we focus on their cardioprotective and antiatherosclerotic effects. According to various in vitro and in vivo studies, the cardioprotective and antiatherosclerotic properties of HT and PC can normalize dyslipidemia, arterial prehypertension and hypertension, diabetes mellitus, oxidative and nitrative statuses, proinflammatory statuses, prothrombotic statuses, endothelial dysfunction, obesity, metabolic syndrome, and mitochondrial dysfunction, modulate the expression of cardioprotective and antiatherosclerotic genes, and reduce the adverse effects of drug treatment, etc. [9,13,14,16,26,31,32,34,37,38,39,40,41,42,43,44,45,46,47,48,49,50,51,52], through multiple pathways [9,13,16,26,31,32,33,34,45,46,47].

In a previous crossover, randomized, double-blind and controlled study, oral supplementation with a combination of HT and PC in middle-aged healthy adults showed anti-atherosclerotic effects by improving endothelial function, blood pressure and levels of circulating oxidized LDL, with more marked improvements in subjects with alterations of these atherosclerotic markers [14]. Data obtained in this clinical trial regarding the effect of this oral supplement on the lipid profile, especially in subjects with dyslipidemia are reported.

## 2. Materials and Methods

The present study was registered at http://clinicaltrials.gov under the number NCT02042742 (access date: 23 January 2014).

### 2.1. Design and Subjects

This was a crossover, randomized, double-blind and placebo-controlled clinical trial, which was conducted over 20 weeks. A full description of the methodology of the study has been previously reported [14]. Briefly, eligible participants were assigned at random to two double-blind 8-week treatment periods receiving the supplement or placebo separated by a washout period of 4 weeks. Participants (*n* = 84) were men and women, aged 45–65 years, recruited at the Nutrition Department of Hospital University La Paz in Madrid, Spain, who voluntarily agreed to take part in the study and gave written consent. Exclusion criteria were body mass index (BMI) ≥30 kg/m^2^, subjects receiving drug treatment for CV risk (e.g., dyslipidemia, hypertension, diabetes mellitus, etc.), presence of family background of premature vascular disease, metabolic syndrome, severe liver or renal dysfunction, cancer, and mental illness or low cognitive ability. Other exclusion criteria were the use of nutritional supplements, intensive physical activity, alcohol use (>30 g/day), and hypersensitivity or allergy to olive and pomegranate by-products. Women still experiencing menstrual cycles were also excluded.

All subjects gave their informed consent to take part in the study, which was approved by the Scientific Research and Ethics Committee of the HULP (Code 3799) in accordance with The Ethical Standards of The Declaration of Helsinki [53].

### 2.2. Intervention and Study Variables

The supplement (SAx) (Pomalive^®^, Euromed S.A., Mollet del Vallès, Barcelona, Spain) (patent **in** concession process) contained 3.3 mg of HT from a standardized olive fruit extract (Mediteanox^®^,) 65 mg of PC from a standardized pomegranate fruit extract (Pomanox^®^ P30) and 331.7 mg of maltodextrin. Identically appearing placebo capsules contained 400 mg of maltodextrin. Subjects were instructed to take three capsules/day of the assigned product (SAx or placebo) with their meals and were instructed to maintain their normal dietary habits. They received the exact number of capsules (in blister packaging) required for each 8-week intervention period (SAx or placebo) during pre-period visits at the study center. Visits were scheduled at baseline and before and after each intervention period. Study variables included the following: (a) diet assessment, (b) anthropometric measurements, (c) vital signs, (d) biochemical analysis of the lipid profile, and (e) compliance with the study products and adverse effects.

The diet was recorded over three days (including one day of the weekend) [20]. Participants registered the weight of foods or, alternatively, cups, spoonfuls, etc., used for household measurements. Records were reviewed by a nutritionist during the study visits in the presence of the participant. The DIAL software (Alce Ingenieria S.L., Las Rozas de Madrid, MD, Spain) was used for the calculation of the energetic and nutritional content of foods and beverages consumed.

Anthropometric data (weight, height, BMI) were collected using standard techniques, adhering to international norms set out by the WHO [54] in the morning by trained personnel with the subject barefoot and wearing only underwear. A bioelectrical impedance analyzer (BIA) was used for estimating body composition (EFG ElectroFluidGraph^®^, Akern S.R.L., Pontassieve, Fl, Italy). Blood pressure and heart rate were measured using a 420 Spot Vital Signs Monitor (Welch Allyn, Skaneateles Falls, NY, USA), determining the mean of three readings.

At the beginning and end of each 8-week intervention period, fasting blood samples were collected for biochemical analysis. Samples were analyzed on the Olympus AU5400 Automated Chemistry Analyzer (Olympus Corporation, Izasa, CA, USA) for levels of total cholesterol, high-density lipoprotein cholesterol (HDL-C), low-density lipoprotein cholesterol (LDL-C) and triglycerides. Results are expressed as mg/dL, values that were considered dyslipidemia were ≥200 mg/dL, ≥150 mg/dL, ≥160 mg/dL, and <50 mg/dL for total cholesterol, triglycerides, LDL-C, and HDL-C levels, respectively.

The participants’ compliance was determined through interviews and a comparison between the number of capsules supplied and that returned in the middle and at the end of each intervention period. When a participant had consumed ≥90% of the capsules supplied, he/she was deemed compliant. Any adverse effects observed during the study were logged. Any unfavorable, unwanted effects (diarrhea, constipation, nausea, vomiting, halitosis, etc.) that were reported by a participant and/or observed by the researchers were defined as adverse effects. No participants showed adverse effects during the study. The participants were informed of their right to leave the study at any time.

### 2.3. Statistical Analysis

In the present clinical trial, a sample size of 38 subjects was determined to be necessary to achieve 90% power (at α = 0.05) with a potential 20% dropout [14,55]. Qualitative data are presented as both counts and percentages. Quantitative data are presented as means ± standard deviations (SDs). The Kolmogorov–Smirnov test was used to assess whether the data were normally distributed, and Levene’s test was used to evaluate the homogeneity of the variances. The denominator’s degrees of freedom were estimated using Satterthwaite’s formula. The possible sequence effects, period effects, and residual effects that can occur in this type of crossover study were analyzed. Multiple comparisons were adjusted for using the Bonferroni method. Two-sided tests were applied, and a *p*-value < 0.05 was considered statistically significant. The statistical analyses were performed using the linear mixed model in the SAS Statistical Analysis Software, version 9.3 (SAS Institute Inc., Cary, NC, USA).

## 3. Results

### 3.1. Recruitment and Study Population

**Figure 1:**
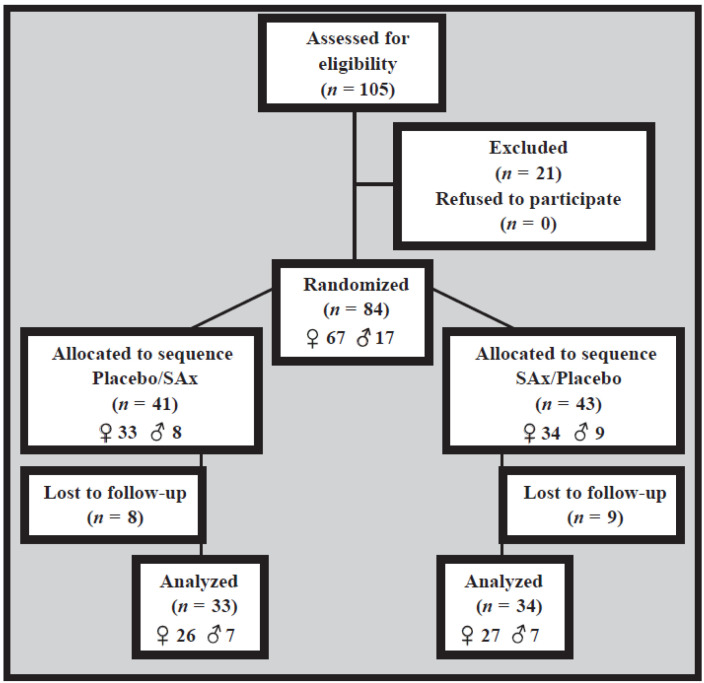
Flow chart depicting the present study.

The present clinical trial was performed between February and June 2013. This study involved 84 apparently healthy subjects (17 males (20.2%) and 67 females (79.8%)) who were found to be suitable for inclusion. There were 17 participants subsequently lost to follow-up (nine in the SAx/Placebo sequence and eight in the Placebo/SAx sequence) for personal reasons (*n* = 15) and noncompliance with the treatment instructions (*n* = 2). As such, 67 participants (14 males (20.9%) and 53 females (79.1%)) completed the present 20-week clinical trial; only their results were included in the statistical analyses (Figure 1).

### 3.2. Baseline Characteristics

Regarding the baseline state in the present clinical trial, there were no significant differences between the participants assigned to the different intervention sequences (Placebo/SAx and SAx/Placebo) in gender, age, smoking habits, anthropometry, lipid profiles, or other variables. The average age of the population was 53.0 ± 4.5 years, and the average BMI was 24.6 ± 3.1 kg/m^2^ (Table 1).

### 3.3. Dietary and Anthropometric Variables

Regarding the results for the dietary and anthropometric variables compared between the beginning and end of the different intervention periods, no significant differences were observed, nor were significant differences found between the different periods in terms of the changes in these variables (Table 2).

### 3.4. Lipid Profile Variables

**Figure 2:**
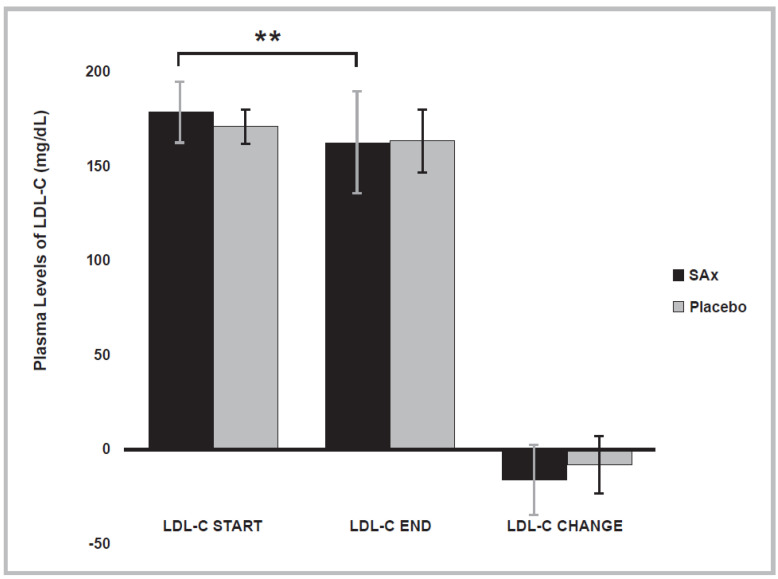
The plasma levels of low-density lipoprotein cholesterol (LDL-C) significantly decreased after SAx treatment (oral supplementation with hydroxytyrosol (HT) and punicalagin (PC)) (black color) in subjects with high plasma levels of LDL-C, a risk factor for CVD, (*n* = 16) (** *p* < 0.01). This effect was not observed after placebo treatment (gray color). The data represent the adjusted means ± standard deviations (SDs) from multivariate models.

Table 3 shows the values obtained for the lipid-profile variables examined. Figure 2 shows a significant reduction after SAx treatment was observed in the plasma levels of LDL-C in subjects with initially high plasma levels of LDL-C (≥160 mg/dL) (SAx period start: 179.13 ± 16.18; end: 162.93 ± 27.05 mg/dL; *p* < 0.004). This significant effect did not occur in these subjects following placebo treatment.

**Figure 3:**
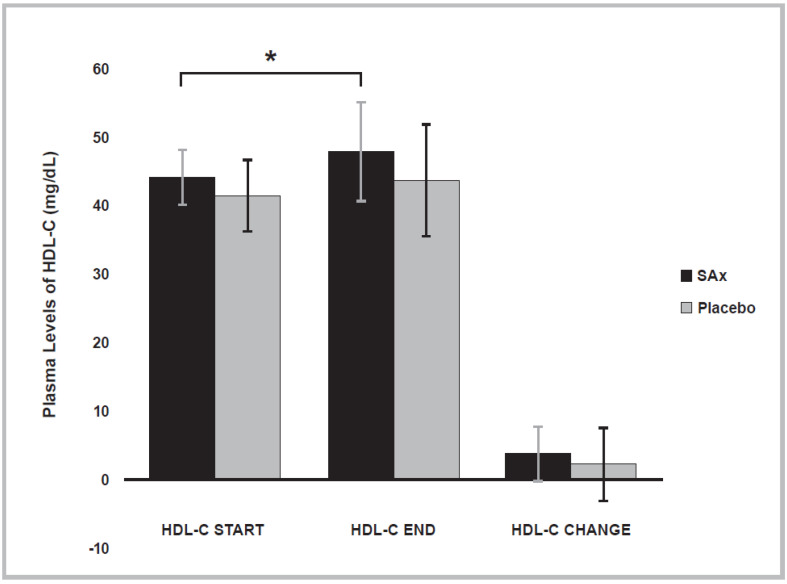
Plasma levels of high-density lipoprotein cholesterol (HDL-C) significantly increased after SAx treatment (oral supplementation with hydroxytyrosol (HT) and punicalagin (PC)) (black color) in subjects with low plasma levels of HDL-C, a risk factor for CVD, (*n* = 8) (* *p* < 0.05). This effect was not observed after placebo treatment (gray color). The data represent the adjusted means ± standard deviations (SDs) from multivariate models.

In addition, at the end of the placebo period, a significant decrease in the plasma levels of HDL-C was observed in the total population (Placebo period start: 64.49 ± 12.65; end: 62.55 ± 11.57 mg/dL; *p* < 0.016). After the SAx period, a significant increase in the plasma levels of HDL-C was observed in subjects with initially low plasma levels of HDL-C (SAx period start: 44.25 ± 3.99; end: 48.00 ± 7.27 mg/dL; *p* < 0.033). After the placebo period, this significant effect on plasma HDL-C levels was not observed in these subjects (Placebo period start: 41.50 ± 5.19; end: 43.75 ± 8.26 mg/dL; *p* < 0.464) (Figure 3). In the present clinical trial, only women showed low plasma levels of HDL-C. There were no men who presented low plasma levels of HDL-C.

**Figure 4:**
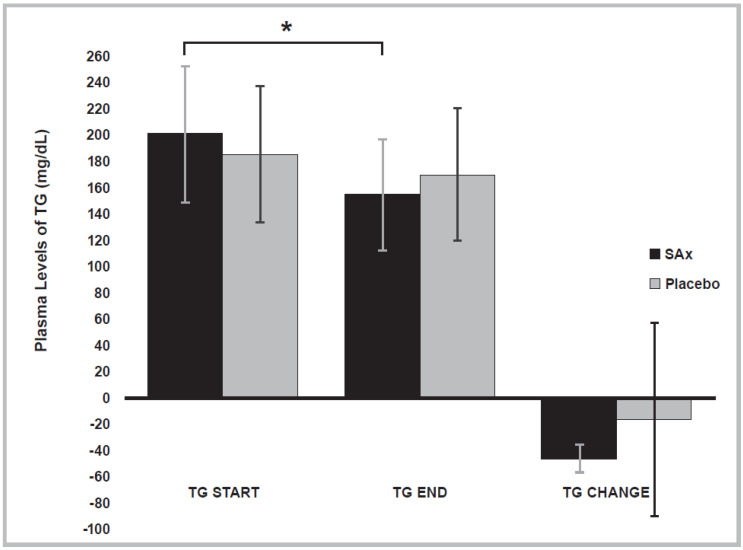
Plasma levels of triglycerides (TG) significantly decreased following SAx treatment (oral supplementation with hydroxytyrosol (HT) and punicalagin (PC)) (black color) in subjects with hypertriglyceridemia, a risk factor for CVD, (*n* = 4) (* *p* < 0.05). This effect was not observed after placebo treatment (gray color). The data repreScheme.

At the end of the SAx period, a significant decrease in the plasma levels of TG was observed in subjects with hypertriglyceridemia (≥150 mg/dL) (SAx period start: 200.67 ± 51.38; end: 155.33 ± 42.44 mg/dL; *p* < 0.017). This significant effect on the plasma levels of TG was not present after the placebo period in these subjects (Placebo period start: 186.00 ± 51.54; end: 170.50 ± 50.32 mg/dL; *p* < 0.700) (Figure 4).

### 3.5. Compliance and Adverse Effects

No significant differences were observed in the numbers of capsules consumed between the different intervention periods or treatment sequences. More than 90% of the capsules provided were consumed by all the participants. No adverse effects derived from the consumption of any treatment were reported.

## 4. Discussion

The present clinical trial is the first study to evaluate the effects of the regular consumption of an oral supplement containing HT and PC on ASVD and CVD markers, such as dyslipidemia (high TC, high LDL-C, low HDL-C, and high TG), in primary prevention in an adult population. The intake of three capsules daily, which contained HT (9.9 mg) and PC (195 mg), for an 8-week period significantly decreased the plasma levels of LDL-C and TG and significantly increased the HDL-C levels in an adult population with dyslipidemia without co-adjuvant treatment, and no adverse effects were observed, even though they frequently occur when using lipid-lowering drugs (e.g., myopathies, renal dysfunction, hepatic dysfunction, rhabdomyolysis, flushing, itching, gastrointestinal irritation, and stomach ulcers) [56,57,58,59,60,61]. In addition, the supplement resulted in a high adherence to the treatment among the participants (>90%). As observed in our previous article, the supplement containing HT and PC produced a significant improvement in ASVD and CVD markers, such as endothelial dysfunction, arterial prehypertension and hypertension (both systolic blood pressure (SBP) and diastolic blood pressure (DBP)) as well as circulating plasma levels of oxLDL [14]. Several studies have shown improvements in dyslipidemia following the intake of HT [9,37,49,62,63,64,65,66,67] or PC [46,68,69,70,71,72,73]. However, most of the studies that have evaluated the effects of these bioactive compounds were performed in vitro or in experimental animals [37,49,50,52,62,63,67,71,74]; only some have involved humans [9,38,64,65,73], and even fewer have evaluated these compounds outside food matrices [14,66,68,75] or studied their combined synergistic effect [14].

An example of this is the study of Cao et al., who observed how dyslipidemia could be prevented by 17-week of supplementation with HT. The study evaluated the effects of various doses of HT (low-dose: 10 mg/kg/day, and high-dose: 50 mg/kg/day) vs. metformin (225 mg/kg/day) in mice with diverse metabolic disorders induced by a high-fat-diet (HFD), or in mice with obesity and type 2 diabetes mellitus (T2DM) (db/db-model mice). Low-dose HT produced significantly decreased fasting glucose levels in the db/db-model mice that were similar to those of the metformin group. In Cao et al.’s study, low- and high-dose HT notably and significantly improved the lipid profile in both the HFD and db/db mice without adverse effects, while metformin did not produce this positive effect in those mice. All the lipid variables were significantly increased under HFD treatment and effectively improved after treatment with both low- and high-dose HT (decreased plasma LDL-C levels (*p* < 0.01), decreased plasma TG levels (*p* < 0.01), reduced plasma free fatty acid levels (*p* < 0.01), increased plasma HDL-C levels and improved LDL-C/HDL-C ratios (*p* < 0.01)). As in our study, due to an increase in the plasma HDL-C levels, a significant reduction in TC was not observed by Cao et al. In addition, HT supplementation could decrease lipid deposits within the livers and muscle tissues of the HFD mice, through the inhibition of the sterol regulatory element-binding protein 1c/fatty acid synthase (SREBP–1c/FAS) pathway, reducing SREBP-1c levels, a well-known regulator of fatty acid and cholesterol synthesis in the liver [52].

The improvement of dyslipidemia with HT has also been reported in other studies, such as the study of Tabernero et al., where they evaluated the effect of HT and its lipophilic derivatives in rats with diverse metabolic disorders induced by a cholesterol-rich diet. The hypercholesterolemic diet was supplemented with 0.04% HT in the different HT groups. After 8-week, a significant reduction in the plasma levels of LDL-C and TC was observed in the HT groups, and there was not a significant decrease in plasma TG [62]. This amelioration of dyslipidemia with HT was also observed in the study of Zhang et al., in which HT was administered at a dose of 10 mg/kg/day orally to mice for 16-weeks. After this period of time, a marked and significant reduction in the plasma levels of the lipid parameters (TC, LDL-C, and TG) and an increase in the plasma levels of HDL-C were observed in the HT group, compared to the control group (by approximately 17.4% (*p* = 0.004), 15.2% (*p* = 0.003), 17.9% (*p* = 0.009), and 26.9% (*p* = 0.033), respectively)). HT improved hepatic steatosis and lipid deposition. The possible pathways for improving lipemia include the regulation of cholesterol metabolism via decreasing the phosphorylation of p38, followed by the activation of AMP-activated protein kinase (AMPK) and inactivation of nuclear factor-kappa B (NF-κB), which, in turn, trigger the blockade of sterol regulatory element-binding protein 2/proprotein convertase subtilisin/kexin type 9 (SREBP2/PCSK9) and the upregulation of low-density lipoprotein receptor (LDLR), apolipoprotein A-I (ApoAI), and ATP-binding membrane cassette transport protein A1 (ABCA1). These steps finally lead to a reduction in LDL-C and an increase in HDL-C in the circulation [67].

A crossover clinical trial with 60 prehypertensive men conducted by Lockyer et al. evaluated the effects of a phenolic-rich olive leaf extract (136.2 mg of oleuropein; 6.4 mg of HT) on lipid profiles, among other variables, during 6-week of treatment, followed by 4-week of washout. In the phenolic-rich olive leaf extract group, after 6-week, the researchers reported a reduction in the plasma levels 308 of TC (−0.32 (±SD 0.70) mmol/L, *p* = 0.002), LDL-C (−0.19 (±SD 0.56) mmol/L, *p* = 0.017), and TG (−0.18 (±SD 0.48) mmol/L, *p* = 0.008)); however, no significant changes were observed in the differences between the study treatments. Although these lipid-lowering effects were obtained in subjects who did not suffer from dyslipidemia, the researchers suggest that the possible mechanisms by which these lipid-lowering effects occur include a decrease in the activities of key cholesterol-regulatory enzymes, such as 3-hydroxy-3-methylglutaryl coenzyme A reductase (HMGR) (the main target of statins) and acyl-coenzyme A: cholesterol acyltransferase (ACAT), resulting in decreased cholesterol biosynthesis, impacting the flow of bile (increasing biliary cholesterol and bile acid concentrations) and leading to its increased fecal excretion [66].

On the other hand, with respect to the results observed with PC on lipid profiles, some authors, such as Kang et al., have observed improvements in dyslipidemia in mice, with these metabolic disorders induced by a HFD. Their study evaluated the effects of administering various doses of PC (low-dose: 10 mg/kg/day, and high-dose: 100 mg/kg/day) to these mice for 12-week. After PC administration, at both doses, there was a significant decrease in the plasma levels of TG, TC, and LDL-C, and a significant increase in those of HDL-C (low-dose: 14%, 16%, 42%, and 19%, respectively, and high-dose: 23%, 25%, 67%, and 35%, respectively) compared with the control HFD (*n* = 6) [46]. A pomegranate leaf extract (PLE) rich in PC produced improvements in the lipid profile in a mouse model in which hyperlipidemia and obesity were induced by a HFD. The treatment group was provided with 400 or 800 mg/kg/day of PLE for 5-week. The results after 5-week were very encouraging. Apart from the improvements in the other parameters evaluated, the study showed a marked and significant reduction in the plasma levels of TC and TG, and a significant improvement in the TC/HDL-C ratio (low-dose: approximately 35% (*p* < 0.01) and 56% (*p* < 0.01), respectively, and high-dose: approximately 29% (*p* < 0.05), 60% (*p* < 0.01), and 24% (*p* < 0.05), respectively)) versus the HFD group (*n* = 11). PLE also significantly attenuated the rise in plasma TG and inhibited intestinal fat absorption in these mice. PLE showed a significant difference in decreasing the appetite of obese mice fed a HFD but showed no effect in mice fed a normal diet [70].

In clinical trials, such as the one conducted by Esmaillzadeh et al., in 22 patients with T2DM and dyslipidemia, 14 women (63.6%) and eight men (36.4%) presented improvements in their lipid profiles after the consumption of 40 g/day of a concentrated pomegranate juice (CPJ) rich in PC for 8-weeks. Although these results showed a significant reduction in the plasma levels of TC (approximately 4%; *p* = 0.006) and LDL-C (approximately 8%; *p* = 0.006), and the LDL-C/HDL-C (approximately 10%; *p* < 0.001) and TC/HDL-C ratios (approximately 6%; *p* < 0.001), there were no significant changes in the plasma levels of TG or HDL-C versus the control group (pre-study period of 8-weeks without CPJ). There were no significant changes between these two periods. The researchers suggested, as a possible mechanism for the improvement of dyslipidemia through the consumption of a CPJ rich in PC, that a reduction in the liver’s levels of cholesterol esters but not an elevation in the fecal excretion of cholesterol or bile acids might affect cholesterol biosynthesis in the liver [73]. Along the same lines are the results observed by Estrada-Luna et al., in women with acute coronary syndrome (ACS), who took a daily dose of 20 g of microencapsulated pomegranate (MiPo) rich in PC for 4-week. After the consumption of MiPo by 11 subjects, there was an improvement in the lipid profiles in the fasting and postprandial conditions, among other parameters evaluated, as evidenced by a significant reduction in the plasma TG (*p* < 0.05; 16%, 8%, and 42% at 0, 4, and 8 h, respectively), TC (*p* < 0.05; between 8% and 15% at all of the three registered times), and LDL-C levels. The most important decrease reported with MiPo treatment was in the plasma LDL-C levels, in both the fasting and postprandial conditions, at any time on the treatment curve (27% in the fasting conditions (*p* < 0.05), and 36% at 4 h (*p* < 0.05) and 35% at 8 h in the postprandial conditions (*p* < 0.05)) compared to those in the pre-supplementation conditions. The fasting plasma levels of HDL-C significantly increased by 11% (*p* < 0.05). These improvements may be due, according to the researchers, to the activation of peroxisome proliferator-activated receptor-α (PPAR-α) and peroxisome proliferator-activated receptor-γ (PPAR-γ), to the overexpression of lipoprotein lipase (LPL) activity, and possibly, to a reduction in the intestinal absorption of TG [68].

The improvements in dyslipidemia observed with the administration of only these bioactive compounds are of great importance since atherogenic dyslipidemia (abnormal changes in the plasma lipid profile, such as a decrease in HDL-C levels and increase in TG and LDL-C levels) is strongly associated with ASVD and the progression of CV complications [76]. The following are among several reasons for this strong association: the elevation of the plasma levels of LDL-C is one of the primary mechanisms in initiating the development of ASVD by inducing its entrance and retention in the arterial intima and leading to extracellular cholesterol accumulation and the formation of cholesteryl ester droplet-engorged macrophage foam cells with transformation to an inflammatory and prothrombotic phenotype in the blood vessels. These major pathways favor the formation of a plaque necrotic core, containing cellular and extracellular debris and LDL-C-derived cholesterol crystals, in addition to increasing the risk of LDL-C’s oxidation to oxLDL [77,78,79,80]. This is a relevant consideration, as high circulating plasma levels of oxLDL is one of the most important markers in the atherogenic process [81], is associated with all stages of ASVD [81], and is a predictor of future CV events, in both CVD-symptomatic subjects [82,83] and apparently healthy or CVD-asymptomatic subjects [79]. A reduction in the plasma levels of HDL-C decreases its antiatherogenic capacity (reverse LDL-C transport, antioxidant effects by inhibiting LDL-C oxidation, vasodilation, anti-inflammatory effects, antithrombotic effects, antiapoptotic effects, vascular endothelial repair, etc.). This highlights the inverse relationship that exists between low plasma levels of HDL-C and CV risk [84], the former behaving as an independent predictor of CVD [84,85,86,87,88]. In addition, an increase in the plasma levels of TG, giving rise to hypertriglyceridemia, is atherogenic through multiple mechanisms, some of which contribute to the formation of lipid deposits in the arterial intima, increasing monocyte activity, stimulating the synthesis of proinflammatory cytokines and procoagulant factors, and promoting endothelial dysfunction [89]. Hypertriglyceridemia also contributes to atherogenesis through its association with other metabolic alterations, such as the reduction of plasma HDL-C levels or the elevation of the plasma levels of small and dense LDL-C, to name a few [89]. In addition, atherogenic dyslipidemia influences the development of important early markers of CV risk, such as endothelial dysfunction [8,90,91,92,93,94] or high circulating plasma levels of oxLDL [94,95,96]. In the development of endothelial dysfunction, there has been speculation that certain mechanisms may be involved, such as the overexpression of the enzyme nicotinamide adenine dinucleotide phosphate (NADPH) oxidase, the activation of c-Jun N-terminal kinase 2 (JNK2) [97,98], an increase in the production of superoxide anion radical (O2−) [98], an increase in the production of asymmetric dimethylarginine (ADMA) [99], and a rise in the circulating plasma levels of oxLDL [100], among other reactive oxygen species (ROS) [100]. On the other hand, subjects with dyslipidemia per se present higher plasma levels of oxLDL than those without these pathologies, as elevated plasma lipid levels are a strong predictor of high circulating plasma levels of oxLDL in diverse types of populations [77,101,102]. Consequently, the described improvements in the plasma lipid profiles of dyslipidemic subjects could contribute significantly to the reduction of endothelial dysfunction and high circulating plasma levels of oxLDL, improvements observed in the current study that we reported in our previous article [14]. One possible limitation of this study is the sample size.

## 5. Conclusions

The daily intake of a supplement containing HT (9.9 mg) and PC (195 mg) for 8-weeks was shown to improve dyslipidemia in an adult population with metabolic disorders. Therefore, the regular consumption of a supplement composed of HT and PC may reduce the CV risks that these subjects face. Further clinical trials are needed to confirm the favorable effects of these polyphenols in humans.

## Figures and Tables

**Table 1:** Baseline characteristics of the participants.

		Placebo/SAx (*n* = 33)	SAx/Placebo (*n* = 34)
Gender	(Female %, *n*)	78.79 (26)	79.41 (27)
Age	(years)	53.21 ± 4.2	52.79 ± 4.8
Smoking	(Smokers %, *n*)	18.18 (6)	26.47 (9)
Weight	(kg)	66.26 ± 11.8	64.08 ± 10.9
BMI	(kg/m^2^)	24.64 ± 2.9	24.56 ± 3.2
Waist circumference	(cm)	80.51 ± 9.2	82.58 ± 9.8
FM	(%)	29.18 ± 6.7	28.76 ± 6.4
FFM	(%)	70.82 ± 6.7	71.24 ± 6.4
MM	(%)	48.03 ± 7.7	47.87 ± 5.5
SBP	(mmHg)	110.3 ± 13.1	110.9 ± 12.9
DBP	(mmHg)	74.06 ± 10.8	73.75 ± 9.5
HR	(bpm)	67.36 ± 8.9	70.41 ± 7.5
TC	(mg/dL)	226.7 ± 29.6	224.6 ± 35.4
LDL-C	(mg/dL)	144.3 ± 23.9	145.3 ± 28.6
HDL-C	(mg/dL)	66.25 ± 12.9	62.00 ± 12.6
TG	(mg/dL)	80.56 ± 24.6	86.52 ± 44.0

Data presented as means ± standard deviations (SDs). SAx: oral supplementation with hydroxytyrosol (HT) and punicalagin (PC); BMI: body mass index; FM: fat mass; FFM: fat-free mass; MM: muscle mass; SBP: systolic blood pressure; DBP: diastolic blood pressure; HR: heart rate; TC: total cholesterol; LDL-C: low-density lipoprotein cholesterol; HDL-C: high-density lipoprotein cholesterol; and TG: triglycerides. There were no significant differences in the baseline state between the two intervention sequences.

**Table 2:** The dietary and anthropometric variables at the beginning and end of the supplementation, and placebo periods.

			SAx (*n* = 67)	Placebo (*n* = 67)
**Energy**	(kcal/day)	Start	1923 ± 513.6	1864 ± 471.9
		End	1891 ± 549.4	1881 ± 569.5
		Change	−31.88 ± 463.2	17.07 ± 355.5
**Carbohydrates**	(%)	Start	38.06 ± 6.5	38.46 ± 8.9
		End	37.62 ± 6.3	39.06 ± 6.5
		Change	−0.439 ± 5.7	0.598 ± 8.1
**Proteins**	(%)	Start	17.24 ± 3.7	17.44 ± 2.9
		End	17.43 ± 3.6	17.60 ± 3.1
		Change	0.193 ± 4.4	0.164 ± 3.5
**Lipids**	(%)	Start	41.42 ± 6.3	40.48 ± 8.8
		End	41.36 ± 5.7	40.19 ± 5.9
		Change	−0.067 ± 5.3	−0.287 ± 8.0
**SFA**	(%)	Start	12.33 ± 2.9	12.46 ± 4.0
		End	12.38 ± 2.8	12.03 ± 2.9
		Change	0.049 ± 2.7	−0.433 ± 4.2
**MUFA**	(%)	Start	19.10 ± 3.6	18.82 ± 4.9
		End	19.56 ± 4.0	19.29 ± 3.5
		Change	0.453 ± 3.8	0.470 ± 4.2
**PUFA**	(%)	Start	6.45 ± 2.3	5.60 ± 1.6
		End	5.98 ± 1.7	5.46 ± 1.8
		Change	−0.470 ± 2.4	−0.134 ± 1.4
**Total Cholesterol**	(mg/dL)	Start	350.5 ± 172.8	303.9 ± 150.4
		End	328.1 ± 133.8	323.0 ± 115.9
		Change	−22.35 ± 210.8	19.04 ± 130.1
**Fiber**	(g/d)	Start	21.95 ± 7.8	21.71 ± 8.0
		End	21.68 ± 8.9	20.47 ± 7.3
		Change	−0.275 ± 8.2	−1.234 ± 6.5
**Weight**	(kg)	Start	65.10 ± 11.3	65.10 ± 11.2
		End	64.93 ± 11.2	64.85 ± 11.3
		Change	−0.173 ± 1.3	−0.249 ± 1.0
**BMI**	(kg/m^2^)	Start	24.58 ± 3.0	24.63 ± 3.0
		End	24.51 ± 3.0	24.48 ± 3.0
		Change	−0.068 ± 0.5	−0.151 ± 0.6
**Waist Circumference**	(cm)	Start	81.85 ± 9.0	81.24 ± 9.8
		End	81.82 ± 9.6	81.25 ± 9.6
		Change	−0.034 ± 2.9	0.008 ± 3.7
**FM**	(%)	Start	29.16 ± 6.6	28.90 ± 6.5
		End	29.56 ± 6.8	29.79 ± 7.2
		Change	0.400 ± 2.8	0.891 ± 3.7
**FFM**	(%)	Start	70.84 ± 6.6	71.10 ± 6.5
		End	70.44 ± 6.8	70.21 ± 7.2
		Change	−0.400 ± 2.8	−0.891 ± 3.7
**MM**	(%)	Start	47.63 ± 6.2	47.52 ± 6.5
		End	46.67 ± 5.7	46.44 ± 5.8
		Change	−0.970 ± 5.1	−1.082 ± 5.8

Data expressed as means ± standard deviations (SDs). SAx: oral supplementation with hydroxytyrosol (HT) and punicalagin (PC); SFA: saturated fatty acids; MUFA: monounsaturated fatty acids; PUFA: polyunsaturated fatty acids; BMI: body mass index; FM: fat mass; FFM: fat-free mass; and MM: muscle mass. In the present clinical trial, no significant differences were observed between the beginning and end of the different intervention periods or in the changes.

**Table 3:** Lipid-profile variables at the beginning and end of the supplementation and placebo periods in population with dyslipidemia.

			SAx (*n* = 67)	Placebo (*n* = 67)
**TC**	(mg/dL) (*n* = 49)	Start End Change	237.6 ± 26.0 234.9 ± 25.1 −2.776 ± 18.8	238.4 ± 20.0 233.0 ± 22.8 −5.388 ± 18.8
**LDL-C**	(mg/dL)	Start	179.1 ± 16.2	171.6 ± 9.1
	(*n* = 16)	End	162.9 ± 27.1 **	163.6 ± 16.9
		Change	−16.20 ± 18.5	−8.063 ± 15.1
**HDL-C**	(mg/dL)	Start	44.25 ± 4.0	41.50 ± 5.2
	(*n* = 8)	End	48.00 ± 7.3 *	43.75 ± 8.3
		Change	3.750 ± 4.0	2.250 ± 5.4
**TG**	(mg/dL)	Start	200.7 ± 51.4	186.0 ± 51.5
	(*n* = 4)	End	155.3 ± 42.4 *	170.5 ± 50.3
		Change	−45.33 ± 10.5	−15.50 ± 73.1

Data expressed as means ± standard deviations (SDs); SAx: oral supplementation with hydroxytyrosol (HT) and punicalagin (PC); TC: total cholesterol; LDL-C: low-density lipoprotein cholesterol; HDL-C: high-density lipoprotein cholesterol; and TG: triglycerides. In the present clinical trial, significant differences were observed between the beginning and end of the SAx period (* *p* < 0.05, ** *p* < 0.01). There were no significant differences in the placebo period or in the changes in the different intervention periods.

## Patent

In concession process.

## Abbreviations

ABCA1	ATP-binding membrane cassette transport protein A1
ACAT	acyl-coenzyme a: cholesterol acyltransferase
ACS	acute coronary syndrome
ADMA	asymmetric dimethylarginine
AMPK	AMP-activated protein kinase
ApoAI	apolipoprotein A-I
ASVD	atherosclerotic vascular disease
BMI	body mass index
CPJ	concentrated pomegranate juice
CV	cardiovascular
CVD	cardiovascular disease
db/db	mice with obesity and type 2 diabetes mellitus
DBP	diastolic blood pressure
FFM	fat-free mass
FM	fat mass
HDL-C	high-density lipoprotein cholesterol
HFD	high-fat-diet
HMGR	3-hydroxy-3-methylglutaryl coenzyme A reductase
HR	heart rate
HT	hydroxytyrosol
HULP	University Hospital La Paz
JNK2	c-Jun N-terminal kinase 2
LDL-C	low-density lipoprotein cholesterol
LDLR	low-density lipoprotein receptor
LPL	lipoprotein lipase
MM	muscle mass
MiPo	microencapsulated pomegranate
MUFA	monounsaturated fatty acids
NADPH	nicotinamide adenine dinucleotide-phosphate
NF-κB	nuclear factor-kappa B
O2-	superoxide anion radical
oxLDL	oxidized low-density lipoprotein
PC	punicalagin
PLE	pomegranate leaf extract
PPAR-α	peroxisome proliferator-activated receptor-α
PPAR-γ	peroxisome proliferator-activated receptor-γ
PUFA	polyunsaturated fatty acids
ROS	reactive oxygen species
SBP	systolic blood pressure
SFA	saturated fatty acids
SREBP-1c	sterol regulatory element-binding protein 1c
FAS	fatty acid synthase
SREBP2	sterol regulatory element-binding protein 2
PCSK9	proprotein convertase subtilisin/kexin type 9
T2DM	type 2 diabetes mellitus
TC	total cholesterol
TG	triglycerides
